# Effect of Yisui Multipurpose Soup's Amelioration on D-Galactose-Induced Neuronal Cells

**DOI:** 10.1155/2022/3372350

**Published:** 2022-06-15

**Authors:** Junfeng Yan, Xia Zhang, Caixian Xiao, Junlu Yi, Qian Chen, Tingliang Gong, Xin Xie, Wenqiang Tao

**Affiliations:** ^1^Department of Endocrinology, Chongqing Traditional Chinese Medicine Hospital, Chongqing 400000, China; ^2^Department of Encephalopathy, Chongqing Traditional Chinese Medicine Hospital, Chongqing 400000, China

## Abstract

This study clarified the regulatory effect of Yisui multipurpose Soup towards D-galactose-induced cognitive impairment cell model on the molecular level. We first constructed and cultured the cell model of cognitive impairment induced by D-galactose in neurons *in vitro* and then cultured the cells in the medium supplemented with different doses of drug-containing serum of Yisui multipurpose soup. Expressions of inflammatory cytokine tumor necrosis factor-*α* (TNF-*α*), inducible nitric oxide synthase (iNOS), nitric oxide (NO), and interleukin-1*β* (IL-1*β*) were assessed by the ELISA and western blot, and cell apoptosis was determined by flow cytometry and TUNEL. The expression changes of apoptosis-related proteins Bcl-2 and Bax were estimated by immunofluorescence, qPCR, and western blot. Finally, we analyzed and made the network interaction diagram of Yisui multipurpose soup-components-targets through the network pharmacology method, from which we could learn that there were 1104 gene targets related to vascular cognitive impairment (VCI) and 1071 component targets of Yisui multipurpose soup. And there were 251 overlapping genes, mainly gathering in protein binding, protein modification, MAPK signaling pathway, and calcium signaling pathway. The expressions of TNF-*α*, iNOS, NO, and IL-1*β* were significantly decreased after the culture medium was replaced by medium containing drug serum. We also found that the effect of high-dose drug-containing serum on the expression of inflammatory factors was better than that of low dose. The Yisui multipurpose soup drug serum in the medium not only significantly increased Bcl-2 expression and effectively reduced Bax expression, but also inhibited the apoptosis of neurons induced by D-galactose. In conclusion, Yisui multipurpose soup could effectively protect D-galactose-induced neuronal cell cognitive impairment by orchestrating expressions of the inflammatory factors TNF-*α*, iNOS, NO, and IL-1*β* and the apoptosis-related proteins Bcl-2 and Bax.

## 1. Introduction

Factors such as cerebrovascular risk factors and clinical or asymptomatic cerebrovascular disease may cause vascular cognitive impairment (VCI), a category of syndrome stemming from dementia caused by mild cognitive impairment [1, 2]. In recent years, the incidence, disability, and mortality rates of VCI have been increasing year by year, and it has developed into the second major dementia disease, which has brought heavy economic burden to patients and their families [3, 4]. Stroke as the third leading cause of death is thought to be a major cause of cognitive impairment. According to the Global Burden of Disease (GBD) data from 1990 to 2013, there were 6.7 million deaths from stroke. And the rate of disability and death from stroke increased by 2% compared with all diseases worldwide [5]. In addition, based on the study of Alzheimer's Society Vascular Dementia Systematic Review Group, there was a high association between stroke and dementia (hazard ratio = 6.1, 95% CI = 3.6 to 10.5), one in twenty stroke victims developed vascular dementia or VCI [6]. Furthermore, there was a 9-fold increased risk of incident dementia after the first ever stroke, and the cumulative incidence was 23% within 10 years [7]. Epidemiological studies in China showed that the incidence of stroke in China was about 274/100,000 per year, and the incidence of VCI after stroke was as high as 81%, among which the incidence of cognitive impairment without dementia was 48.9% and that of dementia was 32.1%. In patients with VCI without mild dementia, 46% of them developed vascular dementia within five years and their fatality was 52% [8–10]. Therefore, enough attention should be paid to patients with VCI and early intervention should be given to improve their quality of life and prolong their survival. However, so far, all treatments have proved ineffective to VCI. Conventional clinical drugs for the treatment of VCI include butylphthalide, nimodipine, and psychotropic drugs, which not only demand a long drug cycle but also hold unclear efficacy and safety. Therefore, the development of high safety VCI drugs for shortening the clinical medication cycle and reducing the side effects of anti-VCI drugs is one of the challenges to the current clinical anti-VCI treatment and rehabilitation [11, 12].

Yisui multipurpose soup was developed by the Department of Encephalopathy of Chongqing Hospital of traditional Chinese medicine based on dialectical theory and treatment theory of traditional Chinese medicine (TCM), applied for cerebrospinal degeneration, demyelination, nutritional metabolism, and other brain demyelination and mental dysfunction diseases. The Yisui multipurpose soup is formulated with 20 Chinese medicinal materials: cooked rehmannia, cornus, astragalus, codonopsis, psoraleae, etc [13, 14]. Individual clinical cases showed that taking the Yisui multipurpose soup could improve the learning and memory of patients with white matter disease. Modern pharmacological studies showed that Rehmannia glutinosa, rhizome, and Amomum glutinosa possess antioxidant and anti-inflammatory properties, while Astragalus membranaceus, Codonopsis pilosula, Cornus officinalis, Salvia miltiorrhiza, oyster, and Schisandra chinensis are endowed with the capabilities of antioxidant, anti-inflammatory, neuroprotective, and lipid metabolism improvement effects. Moreover, ginger has antioxidant, anti-inflammatory, and neuroprotective properties, and bitter cardamom, dodder, Morinda officinalis, silkworm, scorpion, and centipede also have the effect of antioxidant and nerve protection [15, 16]. The curative effect of TCM prescription is based on the principle of the synergistic effect, which makes the combined effect of various traditional Chinese medicines greater than the curative effect of a single medicine. Based on this principle, Yisui multipurpose soup realizes multitargeted combination therapy and plays a more beneficial role in improving cognitive dysfunction than an individual drug.

The cellular biological basis of biological learning and memory is the functional connection and information transmission between neurons or between neurons and other cells. Neuronal cell damage is an important section in the pathogenesis of cognitive disorders, so protecting neuronal function is of great significance for the treatment of cognitive disorders. The symptoms of patients with VCI, such as neuronal loss, neurofibrillary tangles, and activation of glial cells, are mainly originated from the parahippocampal gyrus and then extended to the hippocampus and then gradually spread to other brain regions, and the early onset of VCI might lead to atrophy of the hippocampus. The hippocampal synapse is one of the brain regions most closely related to cognition and most susceptible to changes in the internal and external environment. Its plasticity plays a particularly important role in learning and memory and the development of neurodegenerative diseases. In summary, in light to literature reports and clinical medication experience, we assumed that the Yisui multipurpose soup may be synergistic amid prescription drugs, and the mechanism of action is possibly related to the protection of neuronal cell function. Therefore, we cultured neurons *in vitro* through Yisui multipurpose soup serum to clarify its protective effect and molecular mechanism on neurons.

## 2. Methods

### 2.1. Preparation of Drug-Containing Serum

For drug-containing serum preparation, according to the clinical dosage of Yimedui decoction stipulated in the Chinese Pharmacopoeia 2015 Edition and Experimental Animal Methodology (3rd edition), the optimal dosage for rats is 10 g/kg. Rats fasted for 8 h before administration, and then, they were administered Yisui multipurpose soup by oral gavage once a day for consecutive 7 days. 2 h after the last administration, rats were anesthetized with pentobarbital sodium and blood samples were collected from the abdominal aorta. After incubating at 37°C for 30 min, the serum was separated by centrifugation. The preparation of blank serum was the same as that of drug-containing serum preparation, but rats were administered the same amount of normal saline. The serum of the same group was merged, inactivated at 56°C for 30 min, filtered by using a 0.22 *μ*m microporous membrane, and stored at −20°C for later use [17].

### 2.2. Cell Cultivation

Neurons were purchased from the American model culture Bank and cultured at 37°C in a 5% CO_2_ incubator with DMEM + 15% FBS. The cells were divided into the control group, model group (10 mg/mL D-galactose + PBS), drug-containing serum high-dose group (10 mg/mL D-galactose + 50% drug-containing serum), drug-containing serum low-dose group (10 mg/mL D-galactose + 10% drug-containing serum), and aniracetam group (10 mg/mL D-galactose + 1 mM aniracetam).

### 2.3. The Expressions of TNF-*α*, iNOS, NO, and IL-1 Were Determined by the ELISA

TNF-*α* (#RX302058 R), iNOS (#RX101362H), NO (#RXWB0246), and IL-1 (RX300024 R) ELISA kits were purchased from Quanzhou Ruixin Biotechnology Co., Ltd., China. The procedure was conducted as follows: after balancing at room temperature for 20 min, required laths were taken from the aluminum foil bag, and the remaining laths were sealed with a self-sealing bag and put back to 4°C. Standard wells and sample wells were designated, and different concentrations of standard 50 *μ*L were added to each standard well. Then, 50 *μ*L of the samples to be tested were added into the sample wells, and the blank wells needed no adding. Except for the blank wells, 100 *μ*L of a horseradish peroxidase (HRP)-labeled detection antibody should be added to each well of the standard wells and sample wells, and the reaction wells were sealed with a sealing film and incubated at 37°C in a water bath or incubator for 60 min. When the liquid was discarded, the wet sample wells were gently drained with a suction paper, and the washing solution (350 *μ*L) was added to each well; then, the samples were kept for 1 min, the washing solution was discarded, the wet sample wells were gently drained with the suction paper again, and the plates were washed 5 times repeatedly. After that, 50 *μ*L substrate A and 50 *μ*L substrate B were added to each well, and the samples were incubated at 37°C for 15 min away from light. Then, 50 *μ*L stop solution was added into each well, and the OD value of each well was measured at 450 nm wavelength within 15 min.

### 2.4. Western Blot for Protein Expression Detection

After centrifugation, precooled RIPA lysis solution (#P0013 C, Beyotime, China) was added to the cells, and the cells were placed on ice for lysis for 15 min, followed by whirlpool shock for 30 s, and then placed on ice for lysis for 15 min. Then, the sample was centrifuged at 12,000 r/min at 4°C for 10 min, and the supernatant was taken. The protein samples were quantified by using the bicinchoninic acid (BCA) kit (#P0010, Beyotime, China), and the protein concentration was diluted to 2.5 *μ*g/*μ*L by adding PBS and the sample loading buffer. The protein samples were denatured in boiling water at 100°C for 5 min and then frozen at −80°C for later use. After that, the protein samples were subjected to 10% SDS-PAGE gel electrophoresis, concentrated gel 80V constant pressure electrophoresis for 30 min, and separated gel 120V constant voltage electrophoresis for 90 min. Finally, the proteins were transferred into the PVDF membrane at 70V constant voltage in a humid box for 90 min. Next, the membrane was blocked with Tris-buffered saline with Tween-20 (TBST) solution containing a 5% skim milk powder at room temperature for 1 h; then, the primary antibody (anti-TNF-*α*, 1 : 5000, #ab205587, Abcam, USA; anti-iNOS, 1 : 500, #ab178945, Abcam, USA; anti-IL-1*β*, 1 : 2000, #ab254360, Abcam, USA; anti-GAPDH, 1 : 3000, #bs-0755R, Bioss, China) was added and incubated at 4°C on a slow shaker overnight. On the second day, the PVDF membrane was cleaned 3 times with TBST, 10 min each time. After that, the membrane was added with a secondary antibody for 2 h and then incubated with hypersensitive luminescence chromogenic solution for 1 min. The PVDF membrane was visualized and displayed in the gel imaging system. Finally, the image gray value was analyzed by using ImageJ Software (Rawak Software, Germany).

### 2.5. Apoptosis Detection through Flow Cytometry

The cells were cultured in 6-well plates, and after LPS induction, cells were treated according to grouping requirements and cultured in the incubator. After the cell culture medium was removed, the cells were washed with PBS once and digested with 1 mL trypsin. The cell suspension was centrifuged at 2,000 RPM for 3 min, and then, the cell pellet was collected and gently resuspended with PBS and counted. With the amount of 1 × 10^5^ suspended cells centrifuged at 2,000 RPM for 3 min, the supernatant was discarded, and 195 *μ*L Annexin V-FITC binding solution was added to gently suspend the cells. Then, another 5 *μ*L Annexin V-FITC was added to the solution and mixed gently. The mixture was incubated at 37°C for 10 min away from light and then centrifuged at 2,000 RPM for 3 min, and the supernatant was discarded. 190 *μ*L Annexin V-FITC binding solution was added into the centrifuge tube to gently suspend the cells. An amount of 10 *μ*L PI dye was added into the solution, mixed gently, and placed in an ice bath away from light. Flow cytometry was performed immediately. Annexin V-FITC showed green fluorescence, and PI showed red fluorescence.

### 2.6. Fluorescence TUNEL Detection of Apoptosis

Cells were fixed with precooled 4% paraformaldehyde, fixed at 4°C for 2 h, and washed three times with PBS. Then, the experimental operation was carried out according to the instructions of the TUNEL staining kit (#A111, Nanjing Novizan Co., Ltd., China) and then stained with 4 *μ*g/mL DAPI for 15–30 min in the dark at room temperature. After the staining, we rinsed slides 3 times with PBS and added an antifluorescence quencher to seal the slide. The experimental results were collected by the Guangzhou Mingmei Zhengxiang fluorescence microscopic imaging system.

### 2.7. Immunofluorescence Assay

The slides were washed 3 times with PBS, 5 min each time. 0.5% Triton X-100 was added to the slides, and the slides were drilled at room temperature for 10 min. The slides were immersed in PBS for 3 times, 3 min each time. Ten percent of normal goat serum was added to the slides, and the samples were sealed at room temperature for 60 min. The blocking solution on the slides was absorbed with an absorbent paper, and then, a sufficient amount of a diluted primary antibody was dripped on each slide and the slides were put in a humid box and incubated at 4°C overnight. The wet box was taken out and rewarmed at room temperature for 30 min, and then, the slides were dipped with PBS 3 times, 3 min each time. The excess liquid was absorbed with the absorbent paper, and the diluted fluorescent secondary antibody was added to the slides. The wet box was incubated at 37°C for 60 min. DAPI was dropped on slides and incubated in darkness for 5 min to dye the samples, and then, the slides were washed with PBS 4 times, 5 min each time, to remove the excess DAPI. The slides were sealed with an antifluorescence quench agent and stored in a wet box. The experimental results were collected by the Guangzhou Mingmei Zhengxiang fluorescence microscopic imaging system.

### 2.8. qPCR Assay

Total RNA was extracted using the Trizol method, and a qPCR test was carried out via a T5 Fast qPCR kit (#TSE202, TSINGKE, China). The reaction system was as follows: 2 × T5 Fast qPCR Mix, 10.0 *μ*L; 10 *μ*M forward primer, 0.8 *μ*L; 10 *μ*M reverse primer, 0.8 *μ*L; 50 × ROX reference dye II, 0.4 *μ*L; template DNA, 0.5 *μ*L; and ddH_2_O, 7.5 *μ*L. Reaction conditions were set at 95°C, 30 s; 95°C, 5 s; 55°C, 30 s; 72°C, 30 s; and 40 cycles of reaction. Primer sequences were as follows:

  Bcl-2-F, TGCACCTGACGCCCTTCAC;
  Bcl-2-R, AGACAGCCAGGAGAAATCAAACAG;
  Bax-F, ACAGATCATGAAGACAGGGG;
  Bax-R, CAAAGTAGAAGAGGGCAACC;
  TNF-*α*-F, CATCTTCTCAAAATTCGAGTGACAA;
  TNF-*α*-R, TGGGAGTAGACAAGGTACAACCC;
  GAPDH-F, AGGTCGGTGTGAACGGATTTG;
  GAPDH-R, TGTAGACCATGTAGTTGAGGTCA.

#### 2.8.1. Pharmacological Network Analysis

From the pharmacology of the Chinese herbal medicine system platform (TCMSP) database (http://lsp.nwu.edu.cn/tcmsp.php), we obtained the main ingredients of Yisui multipurpose soup. Then, we selected compounds with drug-like properties DL ≥ 0.18 as the main active ingredients according to the standard of bioavailability OB ≥ 30%, and the possible target genes of each active ingredient were fetched through PharmMapper. In the GeneCards disease database (https://auth.lifemapsc.com/), the disease targets of VCI were obtained, so we could compare the intersection of genes in the two databases and draw the Venn diagram. The interaction network of intersecting genes was analyzed by the functional protein association network database String (http://string-db.org/cgi/input.pl). We used Cytoscape 3.7.1 (https://cytoscape.org/) and DAVID (https://david.ncifcrf.gov/) to performed GO enrichment analysis, KEGG enrichment analysis, drug-target-disease network interaction analysis, etc., of candidate genes [18].

### 2.9. Statistical Analysis

The data were analyzed by statistical software GraphPad Prism 8.0 (GraphPad, USA), and the measurement data were expressed as mean ± SEM. The significance of the differences among groups was determined using one-way ANOVA. *P* < 0.05 was considered statistically significant.

## 3. Results

### 3.1. The Drug-Containing Serum of Yisui Multipurpose Soup Reduced the Expression of Inflammatory Factors

**Figure 1:**
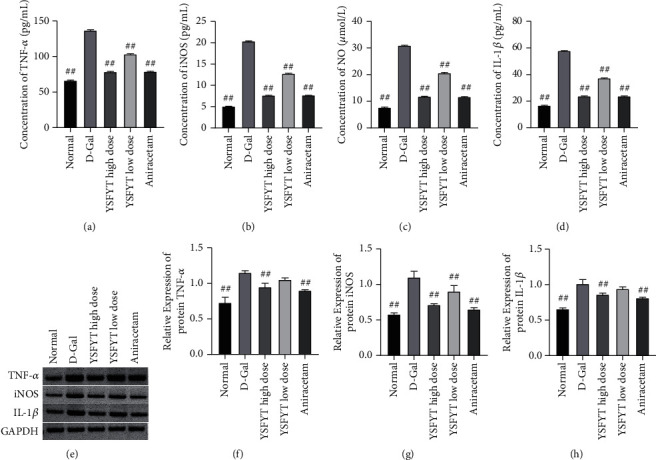
The drug-containing serum of the Yisui multipurpose soup reduced the expression of inflammatory factors. (a) TNF-*α* expression was estimated by the ELISA. (b) iNOS expression was estimated by the ELISA. (c) NO expression was estimated by the ELISA. (d) IL-1*β* expression was estimated by the ELISA. (e) The expressions of TNF-*α*, iNOS, and IL-1*β* were assessed by western blot. (f–h) Gray value statistical results. ##, *P* < 0.01. Normal, normal control group; D-gal, model group; YSFYT high dose, high-dose drug-containing serum group; YSFYT low dose, low-dose drug-containing serum group; aniracetam, positive control group.

This section verified the regulation effect of Yisui multipurpose soup on neuron cells. We constructed the cell model of cognitive impairment by adding D-galactose into cultured neuron cells *in vitro*. At the same time, we also added different doses of drug-containing serum into the cells. Our ELISA results showed that D-galactose significantly increased the release of TNF-*α*, iNOS, NO, and IL-1*β* in neurons, while the expression levels of these molecules were significantly decreased after the addition of drug-containing serum. In addition, we found that the high-dose serum was in possession of a better effect in inhibiting the expression of inflammatory cytokines than the low-dose group, which was similar to the positive control group (aniracetam) (Figures 1(a)–1(d)). Subsequently, we further estimated the expressions of TNF-*α*, iNOS, and IL-1*β* in cells by western blot, and the results were basically consistent with ELISA results, but the difference was that the decreased degrees of TNF-*α* and IL-1*β* in the low-dose serum group were not statistically significant when compared with those of the D-galactose model group (Figures 1(e)–1(h)).

### 3.2. Inhibitory Effect of Drug-Containing Serum of Yisui Multipurpose Soup on D-Galactose-Induced Neuronal Cell Apoptosis

**Figure 2:**
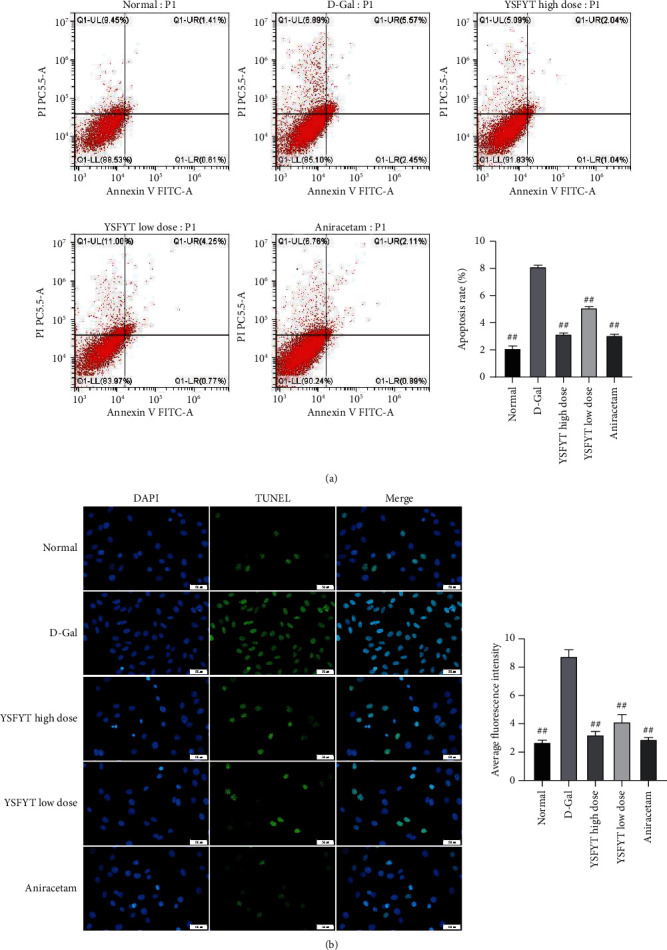
The drug-containing serum of the Yisui multipurpose soup inhibited the neuronal apoptosis induced by D-galactose. (a) The cell apoptosis rate was determined by flow cytometry. (b) Cell apoptosis was evaluated by the TUNEL assay. ##, *P* < 0.01. Scale: 50 *μ*m. Normal, normal control group; D-gal, model group; YSFYT high dose, high-dose drug-containing serum group; YSFYT low dose, low-dose drug-containing serum group; aniracetam, positive control group.

In this section, cell apoptosis was evaluated by flow cytometry. The results showed that the apoptosis rate of the model group (8.02%) was significantly higher than that of the normal control group (2.02%) after D-galactose was added into the model group. After the separate addition of the high-dose drug-containing serum and low-dose serum into the different model groups, the apoptosis rates of neuron cells were 3.08% and 5.02%, respectively, which were all significantly lower than those in the model group (*P* < 0.01) (Figure 2(a)). In addition, the detection of cell apoptosis by fluorescence TUNEL showed that the results obtained were consistent with flow cytometry (Figure 2(b)), which verified the inhibitory effect of the Yisui multipurpose soup serum on D-galactose-induced neuronal cell apoptosis.

### 3.3. Regulation of Yisui Multipurpose Soup towards the Expression of Bcl-2 and Bax

**Figure 3:**
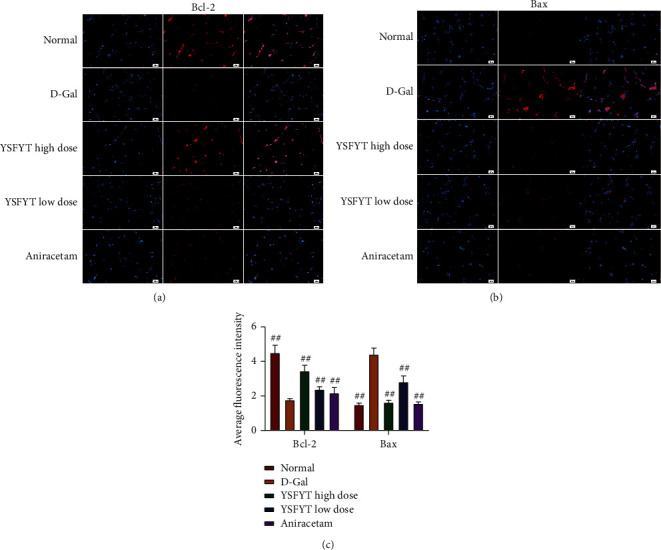
Immunofluorescence was used to determine the regulation of Bcl-2 and Bax expressions in serum of the Yisui multipurpose soup. (a) Immunofluorescence detection on Bcl-2 expression. (b) Bax expression was assessed by immunofluorescence. (c) Statistical results of average fluorescence intensity. ##, *P* < 0.01. Scale: 50 *μ*m. Normal, normal control group; D-gal, model group; YSFYT high dose, high-dose drug-containing serum group; YSFYT low dose, low-dose drug-containing serum group; aniracetam, positive control group.

**Figure 4:**
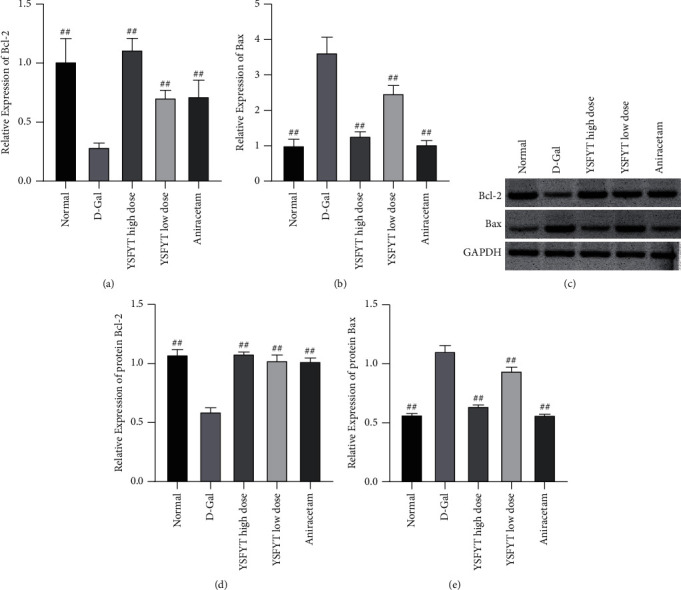
Assays of the qPCR and western blot were used to detect the regulation towards Bcl-2 and Bax expressions in the drug-containing serum of the Yisui multipurpose soup. (a) The qPCR assay for Bcl-2 expression. (b) The qPCR assay for Bax expression. (c) Western blot analysis for Bcl-2 and Bax expressions. d-e Statistical results on the protein gray value. ##, *P* < 0.01. Normal, normal control group; D-gal, model group; YSFYT high dose, high-dose drug-containing serum group; YSFYT low dose, low-dose drug-containing serum group; aniracetam, positive control group.

Finally, by estimating the expressions of antiapoptotic protein Bcl-2 and proapoptotic protein Bax, the mechanism of the Yisui multipurpose soup in the drug-containing serum was further clarified. Cell immunofluorescence detection showed that the expression of Bcl-2 was inhibited after the addition of D-galactose, while the expression of Bax was significantly increased, suggesting that D-galactose is capable of affecting apoptosis-related proteins and leads to neuronal apoptosis. After the addition of the Yisui multipurpose soup serum, the expression of Bcl-2 in the high-dose group was notably increased (*P* < 0.01), while the low-dose group and positive control group had an increasing trend, but there was no significant difference in contrast to the model group. After the addition of the Yisui multipurpose soup serum, expression of Bax was greatly decreased (*P* < 0.01) (Figures 3(a)–3(c)). Subsequently, qPCR and western blot experiments were conducted again to verify the regulation of the expression of Bcl-2 and Bax by the drug-containing serum of the Yisui multipurpose soup. The results showed that the Yisui multipurpose soup serum signally improved the expression of Bcl-2 and sharply reduced the expression of Bax (Figure 4).

### 3.4. Network Pharmacology Analysis onto Yisui Multipurpose Soup

**Figure 5:**
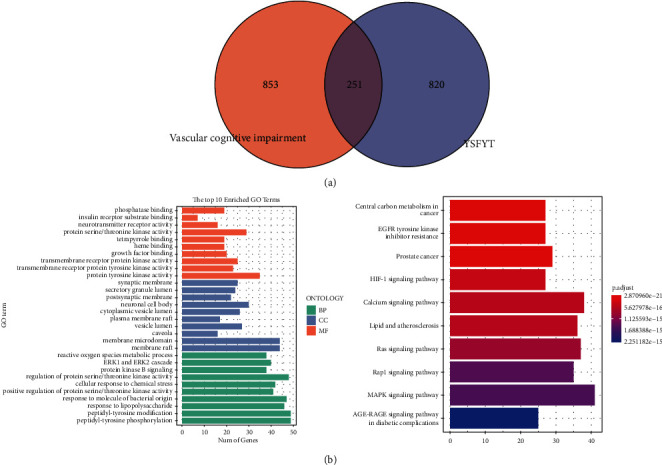
The analysis of targets onto Yisui multipurpose soup and VCI. (a) VCI-related gene targets and the Venn diagram of each component target of the Yisui multipurpose soup. (b) GO analysis and KEGG analysis were performed for the intersection genes of the two.

**Figure 6:**
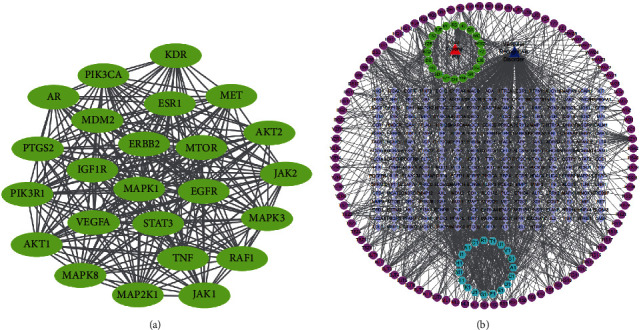
Network pharmacology analysis towards Yisui multipurpose soup. (a) After further screening of enrichment genes, 24 genes with the highest enrichment degree were obtained. PPI analysis was conducted to obtain the interaction between these genes. (b) Yisui multipurpose soup-component-target network interaction diagram.

This study clarified the components of the Yisui multipurpose soup and the regulatory network associated with the targets. We found 1104 VCI-related gene targets in the disease database, 1071 component targets of the Yisui multipurpose soup, and 251 overlapping genes of the two (Figure 5(a)). GO analysis and KEGG analysis were performed on 251 intersected genes, and the results showed that the intersected genes were mainly gathered in protein binding, protein modification, MAPK signaling pathway, and calcium signaling pathway (Figure 5(b)). After deep screening of enriched genes, we found 24 genes with the highest enrichment, which were subsequently analyzed by PPI to reveal the interaction amidst these genes (Figure 6(a)). Finally, according to the obtained data, we drew the network interaction diagram of the Yisui multipurpose soup-component-target (Figure 6(b)).

## 4. Discussion

The biological cognitive process is an intelligent process in which the body recognizes and acquires knowledge, including learning, memory, language, thinking, spirit, emotion, and other behaviors. Cognitive impairment refers to abnormalities in the modules related to learning and memory as well as thinking and judgment of the brain in the process of advanced intelligent processing, resulting in learning and memory dysfunction [19, 20]. Any factors that cause abnormalities in the brain function and structure may lead to cognitive impairment. With the extension of human life span, cognitive decline has gradually become one of the most serious health threats to the aging population, which also brings heavy economic burden and mental pressure to society and family. According to literature reports, the number of dementia patients in China in 2010 was 9.19 million, with a prevalence rate of 9.87‰, among which, 5.69 million patients with Alzheimer's disease (AD) had a prevalence rate of 6.25‰. However, until now, researchers have been concerned about diseases associated with cognitive impairment, such as AD, Huntington's disease (HD), vascular dementia (VD), and Parkinson's disease [21, 22]. The etiology and pathogenesis of PD, are still not very clear, and further research is needed on effective drugs for the treatment and alleviation of cognitive impairment. Through network pharmacology analysis, this study found that there were 1104 VCI-related gene targets, 1071 component targets of the Yisui multipurpose soup, and 251 overlapping genes. Protein binding, protein modification, MAPK signaling pathway, and calcium signaling pathway are mainly involved. The results of this analysis may provide a reference for further research and validation of the molecular regulatory network of the Yisui multipurpose soup.

The mechanism of the galactose-induced cognitive impairment model is that excessive D-galactose-induced metabolites accumulate in cells, resulting in the production of reactive oxygen species and oxidative stress damage to the organism, which simulates the aging process of animals, causing the cognitive function of the organism to decline [23, 24]. The hippocampus is one of the most closely associated regions with learning and memory. And it is one of the few areas in the adult brain where new neurons can be generated, so direct interventions at the neuron level in the hippocampus could yield more meaningful and convincing conclusions about cognitive function [25, 26]. At present, there are few articles on the combined application of D-galactose global and *in vitro* models in the pathogenesis and pharmacodynamics evaluation process; especially, the *in vitro* D-Galactose model for neurons in the hippocampus has received little attention. The D-galactose *in vitro* neuron model can make up for the deficiency of the D-galactose overall animal model in the mechanism study and drug evaluation. In addition, the model construction method is simple; compared with the whole animal model, the *in vitro* model directly acts on neurons in the hippocampal area and the modeling time is shorter, which is more suitable for the study of the mechanism, molecular level, and regulatory pathway [27, 28]. Studies have shown that the recommended dose of 100 mg/kg and modeling time of 8 weeks in the D-galactose overall animal model would not only show an obvious damage effect but also would not cause obvious cognitive decline among experimental animals of different groups, resulting in no obvious phenotypic difference; however, in the D-galactose somatic cell model experiment, the recommended dose is 10 mg/mL and 48 h is the recommended modeling time [29–31]. At this dose and time, cells can be damaged by oxidative stress without obvious morphological changes, which do not affect the detection of subsequent results. In this study, D-galactose would significantly increase the expression of TNF-*α*, iNOS, NO, and IL-1*β* in cultured cells and the apoptosis rate (8.02%) was significantly higher than that of the normal control group (2.02%). Cell immunofluorescence results showed that the expression of Bcl-2 was inhibited after D-galactose supplementation, while the expression of Bax was significantly increased, suggesting that D-galactose can affect apoptosis-related proteins and lead to neuronal apoptosis.

Chinese medicine believes that the lesions of vascular cognitive are present in the brain. The brain controls people's activities, and only when the brain is fully developed, qi and blood are sufficient, and thus, the normal functions of the brain can be guaranteed. When the brain is damaged by falling, brain stasis, blood deficiency, brain displacement, and so on, thus resulting in abnormal brain function and cognitive function. Yangsui means nourishing the brain and the marrow for intelligence because the brain is related to the heart and the kidney. The brain is the sea of the marrow and the center of the human spirit, leading to the spiritual, intellectual, and visceral functions of the human body, and the normal functioning of the brain comes from the nourishment of the kidney [32]. At the same time, the brain and the heart jointly manage the human spirit. The heart and blood can nourish the medulla and make people think quickly. The brain and the Du mai are closely related, and the Du mai is an organic hub within the heart, brain, and kidney. Neurons are the basic units of the structure and function of the central nervous system of the human brain. The information transmission of hippocampal neurons through synapses is the basis of the learning and memory function of the hippocampal area. In this experiment, we selected the pathophysiological changes in cognitive dysfunction in diabetic patients as the most representative ones and the model cells were the basic conditions for the cell model *in vitro* culture. In this study, neurons in the hippocampal region were selected as research subjects. After adding a drug-containing serum, we found that the expression levels of TNF-*α*, iNOS, NO, and IL-1*β* were largely decreased and the effect of the high-dose drug-containing serum on the expressions of inflammatory factors was better than that of the low-dose group. The drug-containing serum of the Yisui multipurpose soup significantly promoted the expression of Bcl-2 and significantly reduced the expression of Bax. Moreover, the drug-containing serum of the Yisui multipurpose soup inhibited the apoptosis of neurons induced by D-galactose. The results of this study are consistent with the basic theory of traditional Chinese medicine. This study has several limitations. Firstly, compared with the whole animal model, to induce VCI on neurons with D-galactose *in vitro* could directly study the functions of the Yisui multipurpose soup. However, the living environment of neurons *in vitro* is not exactly the same as that in organisms. Secondly, we found the MAPK signaling pathway and the calcium signaling pathway are mainly involved in the Yisui multipurpose soup treating VCI by network pharmacology analysis. But these two signal pathways were not verified in this study. At present, the research of traditional Chinese medicine and Chinese medicine also tends to be more in the molecular field; on this basis, the research depth of traditional Chinese medicine and Chinese medicine is also deepening. Based on this study and future studies, we will further introduce new detection methods, such as high-throughput sequencing, intestinal flora analysis, and metabolome analysis in order to comprehensively analyze the mechanism of the Yisui multipurpose soup.

## 5. Conclusion

By treating D-galactose-induced cognitive impairment with the low-dose (10%) and high-dose (50%) drug-containing serum of the Yisui multipurpose soup, the expressions of inflammatory factors TNF-*α*, iNOS, NO, IL-1*β*, and proapoptotic protein Bax were decreased; the expression of antiapoptotic protein Bcl-2 was increased, indicating that the Yisui multipurpose soup effectively protected neuronal cells and reduced the inducing effect of D-galactose on cell apoptosis and the content of inflammatory factors in the cell.

## Data Availability

All data generated and analyzed in the current study are available from the corresponding author on reasonable request.
